# Effect of *in vivo* Hydroxychloroquine and *ex vivo* Anti-BDCA2 mAb Treatment on pDC IFNα Production From Patients Affected With Cutaneous Lupus Erythematosus

**DOI:** 10.3389/fimmu.2019.00275

**Published:** 2019-02-21

**Authors:** Agnes Gardet, Alex Pellerin, Christie-Ann McCarl, Rohan Diwanji, Wenting Wang, Douglas Donaldson, Nathalie Franchimont, Victoria P. Werth, Dania Rabah

**Affiliations:** ^1^Biogen Inc MA, Cambridge, MA, United States; ^2^Department of Dermatology, Perelman School of Medicine, University of Pennsylvania, Philadelphia, PA, United States; ^3^Corporal Michael J. Crescenz VAMC, Philadelphia, PA, United States

**Keywords:** cutaneous lupus erythematosus (CLE), hydroxychloroquine, interferon, systemic lupus erythematosus (SLE), BDCA2 blood dendritic cell antigen 2, toll like receptor (TLR), plasmacytoid dendritic cell, BIIB059

## Abstract

**Objective:** Plasmacytoid dendritic cells (pDCs) are a major source of Type-I Interferon (IFN-I), a key driver in cutaneous lupus erythematosus (CLE). Currently evaluated in Phase II clinical trial, 24F4A (BIIB059) is an antibody targeting BDCA2, an inhibitory receptor expressed on pDCs. Given that Hydroxychloroquine (HCQ), a widely-used CLE therapy, and 24F4A are both able to inhibit pDC-derived IFN-I production; this study aimed to determine whether 24F4A would show an additional inhibitory effect on pDC response after *ex vivo* or *in vivo* treatment with HCQ.

**Methods:** The effect of 24F4A on pDC-derived IFNα was measured from peripheral blood mononuclear cells (PBMC) either from healthy donors in presence or absence of HCQ or from CLE patients clinically exposed to various levels of HCQ. TLR7, TLR7/8, and TLR9 agonists (ssRNA, R848, and CpG-A) were used for pDC stimulation.

**Results:** PDCs were the only producers of IFNα in response to CpG-A, R848, and ssRNA stimulation in PBMC cultures. CLE patients with higher levels of blood HCQ showed lower *ex vivo* pDC responses to CpG-A, but not R848 or ssRNA. In contrast, 24F4A reduced the amount of IFNα produced by pDCs from CLE patients in response to all TLR agonists, irrespective of the blood HCQ level.

**Conclusion:** Our findings reveal that clinically-relevant HCQ concentrations partially inhibit the pDC response to TLR9 and weakly affect the response to TLR7/8 stimulation. 24F4A robustly inhibits pDC responses even in the presence of HCQ, highlighting its unique potential to disrupt pDC disease relevant biology, which could provide additional therapeutic benefit for CLE patients.

## Introduction

Systemic lupus erythematosus (SLE) is a chronic autoimmune disease characterized by autoantibody production, inflammation, and tissue damage in multiple organs resulting from the activation of numerous proinflammatory pathways (1). Cutaneous Lupus erythematosus (CLE) is a skin disorder which can occur as isolated skin manifestation or with concomitant SLE (2, 3). CLE is diagnosed by the clinical morphology of lesions together with a skin biopsy. Based on the skin manifestations, CLE can be classified in three major subtypes: Acute Cutaneous Lupus Erythematosus (ACLE), Subacute Cutaneous Lupus Erythematosus (SCLE) and Chronic Cutaneous Lupus Erythematosus (CCLE), for which the most common form is Discoid Lupus Erythematosus (DLE) (2). CLE is often associated with the presence of autoantibodies (47–97% of the cases are ANA+ and 12–69% are anti-dsDNA+) with varying frequency among ACLE, SCLE, and DLE (4). In addition, CLE commonly presents with skin immune infiltrates and skin immune complex deposition, supporting a role for immune mediated damage in disease pathogenesis (4, 5).

Similar to SLE, IFN-I dysregulation is proposed to be a key pathogenic driver in CLE. Genetic polymorphisms in genomic regions including loci for genes involved in IFN signaling have been associated with higher risk for CLE (6). Additionally, CLE patients often present with IFN signature in blood and in skin lesions with blood IFN signature correlating with skin disease activity score (7–9). Treatment with Sifalimumab (anti-IFNα mAb) and Anifrolumab (anti-IFNAR mAb) showed an improvement of skin disease activity scores in SLE patients presenting with cutaneous disease in Phase II clinical trials further supporting the contribution of IFN-I to the pathogenesis of CLE (10, 11).

Plasmacytoid dendritic cells (pDCs) are specialized cells that robustly secrete IFN-I in response to TLR7/8 and TLR9 stimulation by nucleic acid ligands (12). PDCs accumulate in the skin of CLE patients and account for 5–10% of the immune infiltrate (vs. only 0.1–0.6% of the blood leukocytes) and have been proposed to be the major source of IFN-I in CLE skin lesions (13, 14). Immune complexes (ICs), commonly detected in CLE skin lesions, contain potent TLR ligands that can induce IFN-I production from pDCs (15, 16). Apoptotic debris induced by ultra-violet radiation, a well-known environmental trigger of CLE, leads to the accumulation of DNA and RNA fragments which would also lead to pDC activation and IFN-I production (17). Together, these observations suggest that targeting pDCs in CLE may be an exquisite strategy to effectively impede IFN response in the skin.

Hydroxychloroquine (HCQ), an antimalarial drug, is often used as a first-line therapy in SLE and CLE patients (2). The proposed mechanisms of action of HCQ include the inhibition of endosomal TLRs, antigen presentation, and autophagy all of which are involved in the function of various immune cells (18). Supporting the effect of HCQ on the activation of endosomal TLRs, pDCs from HCQ-treated SLE patients showed impaired ability to produce IFNα in response to TLR9 agonist (19, 20). Although the exact mechanism underlying the effect of HCQ is still unknown, HCQ has been proposed to increase the endosomal intracellular pH, thereby preventing TLR activation of pDCs (21). 24F4A (BIIB059), a monoclonal antibody targeting Blood dendritic cell antigen 2 (BDCA2), is pDC specific therapy currently being evaluated in phase II clinical trials for CLE and SLE (NCT02847598). BDCA2 is a C-type lectin exclusively expressed on the surface of human pDCs and its engagement by 24F4A has been shown to inhibit TLR7- and TLR9-induced production of type-I IFN and other pDC-derived pro-inflammatory mediators (22, 23). Given that 24F4A and HCQ are proposed to impact IFN-I production from activated pDCs, we aimed to ascertain that the effect of 24F4A is not redundant with HCQ. The study described herein demonstrates that 24F4A results in robust inhibition of IFN-I regardless of TLR stimulus or HCQ levels. A clear additive effect on IFN-I inhibition is observed when TLR9 or TLR7/8-activated pDCs are treated with BIIB059 in the presence of HCQ. Taken together, these results highlight the potential added therapeutic benefit of 24F4A when administered with antimalarial compounds such as HCQ.

## Patients and Methods

### CLE Patients

The study received approval by the University of Pennsylvania Institutional Review Board. All patients provided written informed consents. Thirty patients presenting with active SCLE or DLE with or without SLE defined as ≥4 out of 11 ACR classification criteria for SLE were enrolled in the study (24). Patients were required to have at least 3 consecutive visits and documented Cutaneous LE Disease Area and Severity Index (CLASI-A and CLASI-D) scores (25). Patients were excluded if they had no documentation of CLE or of activity or damage scores, if they presented with predominant ACLE or with other skin diseases (e.g., eczema, psoriasis), or if there were enrolled in a clinical study with a drug, biologic, or device for investigational use within 30 days (or 5 half-lives of the agent, whichever was longer) prior to enrollment. Samples were collected at a baseline and a 3 month visit (except for two patients, who did not return for the follow-up visit). Demographics information, disease history, concomitant medications; disease activity scores (SLEDAI-2K and CLASI) were documented throughout the study (26, 27) (Table 1).

**Table 1:** Characteristics of CLE patients included in study.

	**All**	**HCQ < BLQ**	**HCQ**
	***n* = 30**	***n* = 8**	***n* = 22**
Age, years (std)	46.9 (12.78)	44.25 (15.8)	47.2 (11.98)
Female, *n* (%)	20 (67%)	5 (62.5%)	15 (68%)
Caucasian ancestry, *n* (%)	14 (47%)	5 (62.5%)	9 (41%)
Black ancestry, *n* (%)	15 (50%)	3 (37.5%)	12 (55%)
Asian ancestry, *n* (%)	1 (3.3%)	0	1 (4.5%)
Years since CLE diagnostic (std)	10.6 (9.6)	5.5 (8.8)	12.4 (9.6)
Discoid LE, *n* (%)	21 (70%)	5 (62.5%)	16 (73%)
Other chronic cutaneous LE, *n* (%)	4 (13%)	2 (25%)	2 (9%)
Subacute cutaneous LE, *n* (%)	5 (17%)	1 (4%)	4 (18%)
SLE diagnostic, *n* (%)	12 (40%)	1 (12.5%)	11 (37%)
SLEDAI-2K score (std)	4.5 (4.1)	4 (2.4)	4.7 (4.6)
CLASI score (std)	10.6 (8.3)	10.9 (10.8)	10.5 (7.4)
Blood IFN-High, *n* (%)	24 (80%)	5 (62.5%)	18 (82%)
Quinacrine, *n* (%)	10 (33%)	0	10 (45%)
Prednisone, *n* (%)	3 (10%)	1 (12.5%)	2 (9%)
Immunosuppressant, *n* (%)	5 (17%)	1 (12.5%)	4 (18%)

### Healthy Donors

Whole blood samples from healthy donors were used for IFN signature and flow cytometry assays prior to analyses of samples from CLE patients of CLE samples. Blood was obtained through the Blood Donor Program at Biogen after the written informed consents were obtained. All experiments were done in accordance with the IRB protocol 20121572 approved by the Western Institutional Review Board. There was no demographics information available for these donors. Thus, results from these whole blood and assay development experiments were not compared to results from samples obtained from the cohort of CLE patients.

### Whole Blood TLR Stimulation

Human whole blood from healthy donors was treated with either HCQ (Abcam Cambridge, MA, USA) or 24F4A (Biogen, Cambridge, MA, USA) or in combination and incubated for 30 min before treatment with TLR agonists CpG-A (10 μM), R848 (1 μM) or ssRNA (9.2 s ssRNA AGCUUAACCUGUCCUUCAA and A20 control ssRNA AAAAAAAAAAAAAAAAAAAA (4 μg/ml) for 18 h at 37 C and 5% C0_2_ (Sigma-Aldrich St. Louis, MO, USA). Single stranded RNA (200 ng) was complexed with pL-Arginine (360 ng) for 15 min at room temperature in PBS (27). For the purpose of IFNα secretion from human pDCs, 9.2 ss RNA was used as a TLR7 agonist (28). Serum IFNα was measured using VeriKine Human IFNα Multi-Subtype Serum ELISA Kit from Pbl Assay Science (Piscataway, NJ, USA).

### PBMC Preparation

Whole human blood was collected in sodium heparin tubes. PBMC were isolated using a ficoll plaque plus gradient, washed twice with PBS containing 2 mM EDTA and 0.5 % BSA and cryopreserved in 90% FBS and 10%. After thawing, cells were maintained in RPMI 1640 media containing 10% FBS, HEPES, penicillin, streptomycin, L-glutamine, and non-essential amino acids.

### PBMC TLR Stimulation

PBMC (0.5–2 × 10^6^ cells) were treated with 24F4A, isotype control mAb, or culture media for 30 min before treatment with TLR agonists CpG-A 10 μM, R848 1 μM (Invivogen San Diego, CA, USA), or 4 μg/ml of ssRNA complexed with pL-Arginine at 37C and 5% C0_2_. After 2 h, cells were treated with 5 μg/ml Brefeldin A for 4 h before staining.

### FACS Staining

Cell staining was performed using anti-BDCA2 mAb 24F4A-AlexFluor 647 (Biogen, Cambridge, MA, USA), APC-H7 anti-HLADR mAb (L243), V500 anti-CD14 mAb (M5E2) and PE-CF594 anti-CD3 (SP34-2), PE-Cy7 anti-CD123 mAb (7G3) and FITC anti-CD20 mAb (2H7) from BD Biosciences (San Jose, CA, USA), PE anti-IFNα (LT27:295) from Miltenyi Biotech (Somerville, MA, USA) and PerCP/Cy5.5 or BV421 anti-BDCA4 (12C2), FITC anti-TNFα (mAb11), PE anti-IL6 (MQ2-13A5) from Biolegend (San Diego, CA, USA).

Staining was performed at 4°C in PBS containing 2% FBS. Cells were incubated with 10 μg/ml human Fc block (BD Biosciences, San Jose, CA, USA) for 20 min before washing cell staining for 30 min. Foxp3/Transcription Factor Staining Buffer Set (eBioscience, Waltham, MA, USA) was used as per manufacturer's recommendations for intracellular IFNα staining. Fluorescence activated cell sorting (FACS) was performed using BD LSR-II flow cytometer (BD Biosciences, San Jose, CA, USA) prior data analysis with FlowJo software v10 (Treestar, Ashland, OR, USA) and GraphPad Prism7 (La Jolla, CA, USA). To ensure high confidence in the pDC response data, we excluded samples that showed <100 events in the pDC gate. This led to differences in the numbers of donors for each analysis.

### Blood HCQ Levels

Hydroxychloroquine levels were measured in whole blood samples collected in K2/EDTA tubes using liquid chromatography tandem-mass spectrometry after acetonitrile protein precipitation. Control analytes HCQ and d4-HCQ were obtained from US pharmacopeia Convention (Rockville, MD, USA) and Santa Cruz Biotechnology (Dallas, TX, USA), respectively. The high-performance liquid chromatography system consisted of LC-20-ADXR prominence pumps and a SIL-20ACXR prominence autosampler with rack changer (Shimadzu, Kyoto, Japan) and Atlantis HILIC Silica 3uM column (Waters, Milford, MA, USA). Detection was performed used an API 5500 triple quadrupole mass spectrometer (Sciex, Framingham, MA, USA).

### Blood IFN Signature Score

Blood IFN signature scores were determined using a gene set previously used as a blood IFN biomarker gene set in clinical trial (29). Gene expression levels from IFN-responsive genes (*IFI6, IFI27, IFI44, IFI44L, RSAD2*) and housekeeping genes (*ACTB, GAPDH, UBC, YWHAZ*) were determined using RT-qPCR. Total RNA was extracted from whole blood Paxgene tubes. RNA was reverse-transcribed using High Capacity cDNA Reverse Transcription Kits (Life Technologies, Carlsbad, CA, USA) before qPCR analysis using 96 × 96 chips on the Fluidigm BioMark HD real time PCR analyzer. The delta-cycle threshold (ΔCt) value of a gene was calculated by using the raw Ct value of each gene minus the mean Ct of the housekeeping genes for a given sample. The final ΔCt normalized gene expression level of that gene was calculated as 10-ΔCt. The arithmetic mean of the final normalized ΔCt for a given set of IFN inducible genes was calculated and designated as the IFN signature score.

## Results

### 24F4A Inhibits Whole Blood IFNα Production in the Presence of HCQ After CpG-A, R848, or ssRNA Stimulation

**Figure 1:**
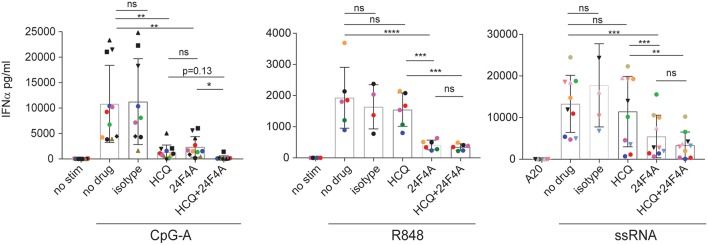
Effect of HCQ and BIIB059 on IFNα release from human whole blood stimulated with CpG-A, R848, or ssRNA. Whole blood from human healthy donors was stimulated or not with CpG-A (10 μM), R848 (1 μM), or ssRNA (4 μg/ml) complexed with pARG in presence or absence of HCQ (550 ng/ml), isotype control mAb (10 μg/ml), 24F4A (10 μg/ml) or a combination of both HCQ and 24F4A. Secreted IFNα was measured 18 h after stimulation in the serum using ELISA. Same color and/or shape-coded data points represent data obtained from a given donor (*n* = at least 6 donors). Statistical tests were performed using paired one-way Anova (ns, non-significant *p* ≥ 0.05, *p < 0.05, **p < 0.01, ***p < 0.001, ****p < 0.0001).

First we wanted to determine whether treatment with 24F4A would lead to additional inhibition of IFNα production from pDCs in the presence of HCQ. Whole blood from healthy donors was stimulated with various endosomal TLR ligands; TLR9 agonist CpG-A, TLR7/8 agonist R848 and TLR7 agonist 9.2 ssRNA and treated with a clinically relevant range of concentrations of HCQ (6.9–5000 ng/mL) (30–32), with or without 24F4A. A HCQ concentration of 555 ng/mL represents the average HCQ blood exposure in the cohort of CLE patients studied. At this concentration HCQ reduced the mean CpG-A- induced IFNα level from 10,800 pg/mL (±2,286) to 1,300 pg/mL (± 424), while having no statistical impact on the mean level of R848 or ssRNA induced-IFNα (Figure 1). Treatment of whole blood with a clinically relevant concentration of 24F4A (10 μg/mL) (33) reduced IFNα levels after stimulation with all 3 agonists (Figure 1, Supplementary Figure 1A). Additionally, the residual TLR agonist- induced IFNα in the presence of 555 ng/mL HCQ was further reduced with the treatment of 24F4A (Figure 1).

We also tested doses of HCQ above 555 ng/mL with all 3 agonists in the presence of 24F4A. HCQ was consistently more potent at inhibiting CpG-A-induced IFNα compared to R848 or ssRNA (Supplementary Figure 1A). Only at high doses (1,666 and 5,000 ng/ml), HCQ was able to achieve >50% inhibition of R848-induced IFNα and had a minimal effect on ssRNA-induced IFNα (Supplementary Figure 1A). Of note, the effect of HCQ treatment on IFNα production was more consistent across donors following CpG-A and R848 stimulation than ssRNA (Supplementary Figure 1B, Figure 1). It is unclear why the effect of HCQ on pDC response after ssRNA stimulation showed a large variability across donors. This was not related to possible differences in ssRNA-pARG complex between experiments since different donors were tested on the same day using the same ssRNA preparation and we still observed variability in HCQ mediated IFNα inhibition (Supplementary Figure 1A). Regardless of the effect of HCQ, additional treatment with 24F4A led to consistent inhibition of IFNα across donors after stimulation with all the TLR agonists used (Figure 1, Supplementary Figure 1A).

Together these data indicated that, *in vitro*, clinically-relevant concentrations of HCQ are more effective at inhibiting CpG-A-induced IFNα from whole blood compared to R848 and ssRNA induced-IFNα. Moreover, these results highlight that 24F4A further reduces residual IFNα production in the presence of HCQ after stimulation with any of these TLR agonists.

### HCQ-Mediated Inhibition of IFNα Production by pDCs Can Be Measured by Flow Cytometry in PBMC Isolated From HCQ-Exposed Whole Blood Samples

We next wanted to confirm that 24F4A can lead to additional inhibition of pDC responses in HCQ-treated CLE patients. As measurement of total release IFNα from PBMC by ELISA could be affected by patient-to-patient variability in pDC frequencies, a flow cytometry assay was developed to measure intracellular IFNα production by pDCs. Using flow cytometry, we detected robust intracellular staining of IFNα in stimulated pDCs, (Supplementary Figure 2A), while an isotype control antibody did not display any background staining (data not shown). PDCs (BDCA2+, BDCA4+, CD123+) were the only source of IFNα upon CpG-A, R848, or ssRNA stimulation in the PBMC culture. The concentrations of CpG-A (10 μM), R848 (1 μM) or ssRNA (4 μg/mL) were chosen based on the optimal IFNα response induced in PBMC as measured by flow cytometry and ELISA (Supplementary Figures 2B,C). Using PBMC from at least 2 healthy donors for each TLR agonists, we confirmed that fresh and cryopreserved PBMC from the same donor had similar IFNα positive pDCs upon stimulation (data not shown).

**Figure 2:**
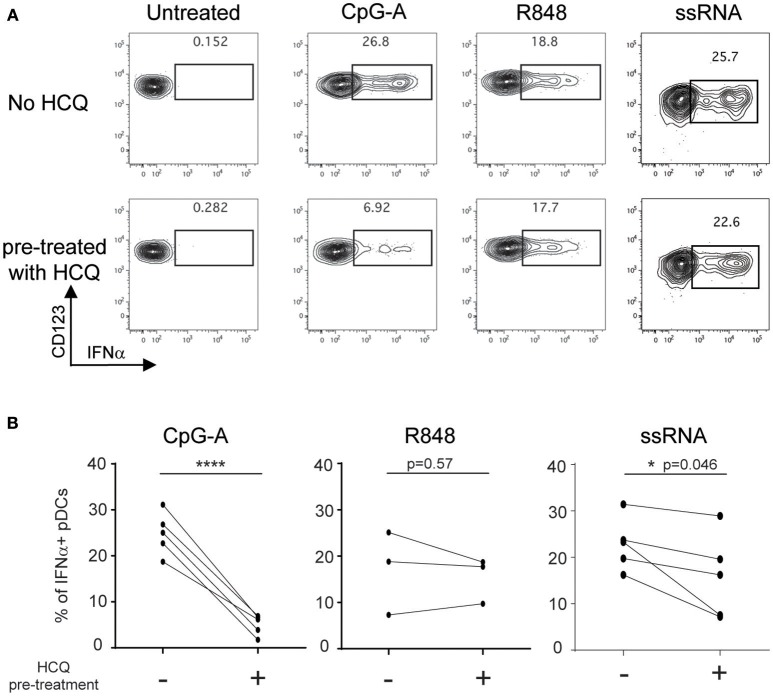
HCQ-mediated inhibition of IFNα production from pDCs in response to CpG-A and ssRNA, but not to R848, can be detected after isolation of PBMC from whole blood pre-treated with HCQ. Whole blood samples from healthy donors were treated with HCQ (1000 ng/ml) or not for 1 h prior to PBMC isolation and then stimulated with CpG-A (10 μM), R848 (1 μM) or ssRNA (4 μg/ml) for 6h. IFNα was analyzed by intracellular cytokine staining. **(A)** Representative dot plots of IFNα+ cells within a BDCA4+ and CD123+ gate. **(B)** Percentages of IFNα-producing pDCs detected as shown in **A**) after CpG-A stimulation (*n* = 5 donors), R848 stimulation (*n* = 3 donors), or ssRNA (*n* = 5 donors) from PBMC isolated from whole blood pre-treated with HCQ or not. Statistical significance was assessed using two-tailed paired Student's t-test (*p < 0.05, ****p < 0.0001).

HCQ accumulates in the red blood cells and leukocytes in the blood of treated patients (34). Therefore, we needed to ascertain that the effect of HCQ on IFNα production persisted after PBMC isolation from whole blood which requires both RBC removal and washing out of serum HCQ. To this end, whole blood samples from healthy donors were treated with 1,000 ng/ml of HCQ for 1 h prior to PBMC isolation and then cryopreserved to mimic the conditions of the cryopreserved PBMC samples to be analyzed from CLE patients. PBMC were then thawed and stimulated with CpG-A, R848, or ssRNA. Under these conditions, HCQ led to inhibition in the percentage of IFNα positive pDCs upon CpG-A and ssRNA stimulation (mean inhibition 79.4% ± STD 9.6, *n* = 5, *p* = 0.0006 and 33.3% ± STD 24.6, *n* = 5, *p* = 0.046), while it did not significantly affect the IFNα response elicited by R848 (Figures 2A,B).

Taken together, these data demonstrate that the flow cytometry assay developed is an appropriate technical approach to evaluate samples from CLE patients and confirm that PBMC collected from HCQ-treated CLE patients retain the HCQ-mediated inhibition of IFNα production from pDCs.

### High Blood HCQ Levels in CLE Patients Lead to Reduced IFNα Production From pDCs Following Stimulation With TLR9, but Not TLR7/8 Agonists

**Figure 3:**
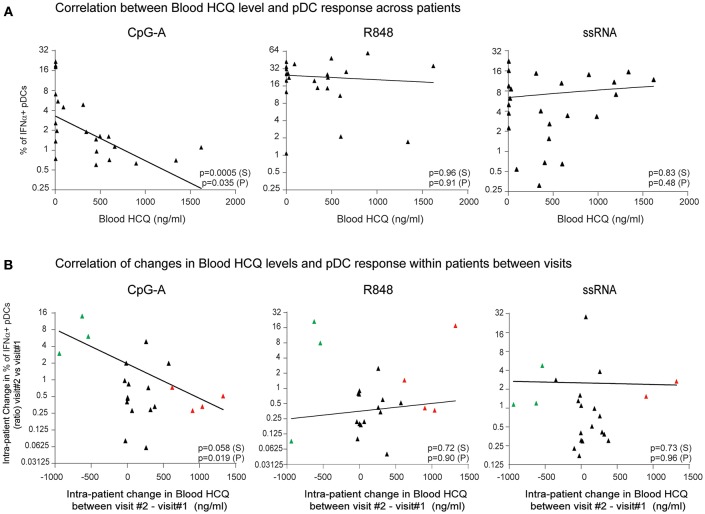
The percentage of IFNα-producing pDCs upon stimulation with CpG-A, but not R848 or ssRNA, is negatively correlated with whole blood HCQ concentrations from CLE patients. **(A)** Association of the percentage of IFNα-producing pDCs identified by flow cytometry from PBMC after CpG-A (*n* = 22 donors), R848 (*n* = 22 donors), or ssRNA (*n* = 24 donors) stimulations with the HCQ concentrations in whole blood from CLE patients. Patients with non-detectable blood HCQ levels are shown at 0 ng/ml blood HCQ **(B)** Association of the change (ratio) between 3-month apart clinical visits in the percentage of IFNα-producing pDCs identified by flow cytometry from PBMC after CpG-A (*n* = 21 donors), R848 (*n* = 21 donors), or ssRNA (*n* = 22 donors) stimulation with the change in the blood HCQ concentration of CLE patients. Data points from patients with increase in blood HCQ concentration >600 ng/ml between the two visits are highlighted in red. Data points from patients with decrease in blood HCQ concentration >500 ng/ml between two visits are highlighted in green. Statistical association was assessed using Pearson's (P) and Spearman's rank (S) correlations.

To determine whether clinical HCQ exposure in CLE patients could affect TLR9 and TLR7/8-induced IFNα production, whole blood samples and PBMC were obtained from 30 CLE patients at baseline visit (Table 1). After exclusion of flow cytometry data that did not pass quality control criteria (see Methods), data was available for at least 22 patients at baseline visits and at least 21 patients at both baseline and the 3 months visits, depending on the TLR agonist (Figure 3). At the baseline visit, the percent of CpG-A-induced IFNα positive pDCs was negatively correlated with the levels of blood HCQ in CLE patients (Spearman's correlation *p* = 0.0005) (Figure 3A). Longitudinal analysis showed that the ratio of percentages of IFNα positive pDCs upon CpG-A stimulation from visit 2 to visit 1 was negatively correlated with the change in blood HCQ concentrations between visit 2 and visit 1 (Figure 3B) (Spearman's correlation *p* = 0.0584). For instance, patients with increased blood HCQ concentrations of more than 600 ng/ml between the two visits showed an average decrease of 2-fold in the percent of CpG-A-induced IFNα positive pDCs between the 2 visits (*n* = 4, highlighted in red triangles in Figure 3B). Conversely, patients with decrease blood HCQ concentrations of more than 500 ng/ml between the 2 visits showed an average increase of 6-fold in the percentage of CpG-A-induced IFNα positive pDCs (*n* = 3, highlighted in green triangles in Figure 3B). In contrast, there was no statistically significant correlation between the blood HCQ levels and R848 or ssRNA-induced IFNα positive pDCs from cross-sectional analysis or the longitudinal analysis (Figures 3A,B).

In summary, the data from both cross-sectional and longitudinal analyses obtained from CLE patients on HCQ, indicated that HCQ therapy in CLE patients inhibits the pDC-mediated IFNα response to a TLR9 agonist, while there was no effect of HCQ on TLR7/8-induced IFNα production from pDCs.

### 24F4A Treatment Reduces *ex vivo* IFNα Production From pDCs From CLE Patients Receiving HCQ Therapy

**Figure 4:**
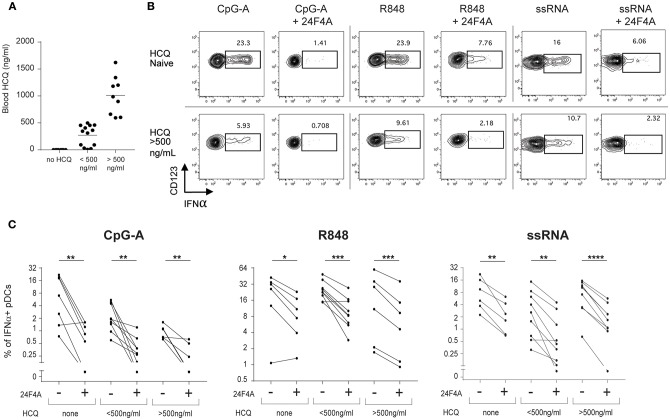
24F4A further reduces pDC IFNα production after CpG-A, R848 or ssRNA stimulations of PBMC isolated from CLE patients regardless blood HCQ levels. **(A)** Concentrations of HCQ in whole blood from the CLE patient cohort studied (*n* = 30). **(B)** Representative dot plots of IFNα+ cells within a BDCA4+ and CD123+ gate after CpG-A, R848 or ssRNA stimulation with or without pre-treatment with 24F4A (10 μg/ml 30 min) from CLE patient without detectable blood HCQ (top panel) or a CLE patient with a blood HCQ concentration of more than 500ng/ml (bottom panel). **(C)** Effect of 24F4A on the percentage of IFNα-producing pDCs induced by CpG-A, R848 and ssRNA stimulations and detected by flow cytometry in PBMC from CLE patients without detectable level of blood HCQ (*n* ≥ 6 donors), with blood HCQ concentrations lower than 500 ng/ml (*n* ≥ 8 donors) or >500ng/ml (*n* ≥ 6 donors). Statistical significance was assessed with a two-tailed paired Student's t-test using log2-transformed values (*p < 0.05, **p < 0.01, ***p < 0.001, ****p < 0.0001).

Previous *in vitro* experiments with whole blood from healthy donors demonstrated that 24F4A could further decrease IFNα production in the presence of HCQ (Figure 1). Therefore, we wanted to investigate whether 24F4A would further impact pDC-derived IFNα from CLE patients that received HCQ therapy (*n* = 30) (Table 1). HCQ concentrations were measured at baseline in whole blood samples of the CLE patients enrolled in the study (Figure 4A). The mean of the whole blood HCQ concentration was 573 ng/ml and the median was 456 ng/ml. PBMC from CLE patients were treated *ex vivo* with 24F4A and subsequently stimulated with CpG-A, R848, or ssRNA prior to flow cytometry assays to measure the percentage of IFNα+ pDCs (Figure 4B). Since a concentration of blood HCQ of <500 ng/ml is typically considered as sub-therapeutic (30–32), three groups of patients were analyzed based on their blood HCQ levels: no detectable blood HCQ (*n* ≥ 6 patients), blood HCQ <500 ng/mL (*n* ≥ 8 patients) and blood HCQ > 500 ng/mL (*n* ≥ 6 patients) (Figure 4C). Note that the number of patients vary per group for each TLR agonist stimulation due to exclusion of samples with low pDC events (see methods and figure legends). We confirmed that *ex vivo* CpG-A-, R848-, and ssRNA-induced IFNα from pDCs could be inhibited (~65–75, 45–55, and 65–75%, respectively) by treatment with 10 μg/ml 24F4A irrespective of the patient's blood HCQ levels (Figure 4C, Supplementary Table 1). Treatment with an isotype control at the same concentration had no impact on percent of IFNα+ pDCs (Supplementary Figure 3). In our cohort, there were 10 patients receiving HCQ and quinacrine concomitantly. Of these, we obtained data for at least 8 patients that passed the quality control criteria for all stimuli tested. In patients where HCQ exposure was <500 ng/mL, 24F4A treatment led to a significant reduction in IFNα under all stimulation conditions in presence of quinacrine (Supplementary Figure 4).

**Figure 5:**
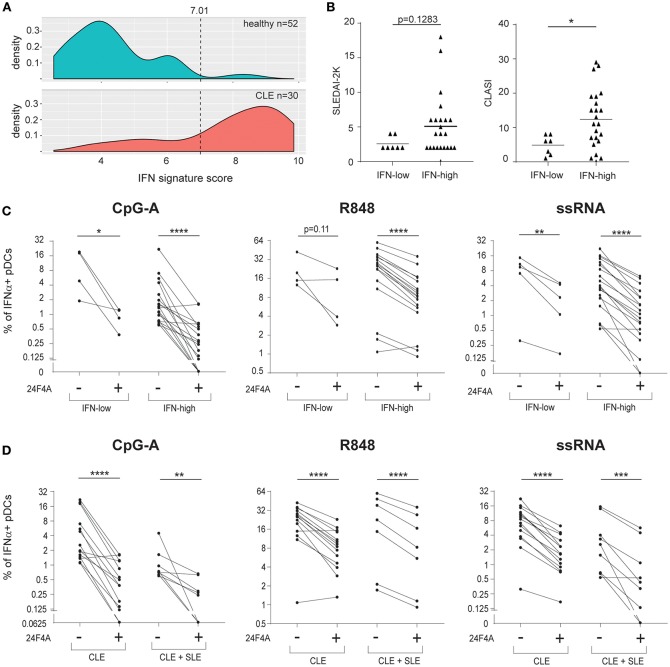
24F4A further reduces pDC IFNα production after CpG-A, R848 or ssRNA stimulations of PBMC isolated from CLE patients with blood IFN-high signature or concomitant SLE diagnostic. **(A)** Distribution of the blood IFN signature scores in healthy individuals (*n* = 52) and CLE patients (*n* = 30). **(B)** Comparison of SLEDAI2-K disease scores (left) or CLASI skin scores (right) between CLE patients with blood IFN-low and blood IFN-high signature scores. Statistical significance was assessed using Mann-Whitney test (*p < 0.05). **(C)** Effect of 24F4A on the percentage of IFNα-producing pDCs induced by CpG-A, R848 and ssRNA stimulations and detected by flow cytometry in PBMC from CLE patients with blood IFN-low (*n* = 4 donors) or IFN-high signature (*n* ≥ 17 donors). Statistical significance was assessed with a two-tailed paired Student's *t*-test using log2-transformed values (*p < 0.05, **p < 0.01, ****p < 0.0001). **(D)** Effect of 24F4A on the percentage of IFNα-producing pDCs induced by CpG-A, R848 and ssRNA stimulations and detected by flow cytometry in PBMC from CLE patients with or without concomitant SLE diagnostic (*n* ≥ 7 donors and *n* ≥ 14 donors, respectively). Statistical significance was assessed with a two-tailed paired Student's *t*-test using log2-transformed values (**p < 0.01, ***p < 0.001, ****p < 0.0001).

In addition to measuring blood HCQ levels of the CLE patients, we also measured whole blood IFN signature scores to explore whether the effect of 24F4A may be different in patients with IFN-low vs. IFN-high signature. The Blood IFN signature was determined using gene expression analysis by qPCR for a set of IFN-responsive genes previously used as a biomarker in clinical study (29). IFN-low signature range was defined using results from 52 healthy donors (Figure 5A). From our study, 80% of the CLE patients showed high IFN blood scores (Table 1). Similar to previous reports from SLE and CLE cohorts (7, 35, 36), the IFN-high patient group presented with a higher skin activity score and tended to have a higher disease activity score based on SLEDAI 2K (*p* < 0.05 and *p* = 0.1283, respectively, Figure 5B). 24F4A significantly reduced the percentage IFNα+ pDCs in response to CpG-A, R848, and ssRNA stimulation in blood IFN-high signature patients (Figure 5C). Statistical significance was achieved with CpG-A and ssRNA stimulation in blood of IFN-low signature patients, but not with R848 stimulation, *p* = 0.11 (Figure 5C). Power analysis revealed that an additional 6 patients would have been necessary to detect a percentage inhibition of 50% with power of 0.8 with a two-tailed paired Student's *t*-Test, assuming that the biological variation would be the same between the IFN-low and IFN-high patients.

Additionally, our data showed that 24F4A was able to inhibit IFNα from CpG-A, R848, and ssRNA stimulated PBMC from the cohort of CLE patients studied regardless of the presence of systemic disease (SLE) (Figure 5D).

Taken together, these data highlight that, while 24F4A and HCQ could potentially impact similar pDC biology, 24F4A is able to strongly impede *ex vivo* IFNα production from CpG-A, R848, and ssRNA stimulated pDCs from CLE patients regardless of clinical HCQ levels, IFN signature, or presence of systemic disease.

## Discussion

Hydroxychloroquine is approved for the treatment of malaria, and rheumatic diseases such as SLE, CLE, rheumatoid arthritis (RA), and is also commonly used for the treatment of cutaneous lesions of dermatomyositis (DM). The rheumatic diseases for which HCQ seems to provide clinical benefits exhibit systemic and disease target tissue IFN-I molecular signatures (37, 38). Multiple studies have reported that patients with high disease activity scores (SLEDAI score in SLE, CLASI score in CLE and CDASI score in DM) tend to have higher IFN signature (7, 35, 36, 38–40), highlighting a relationship between IFN-I activity and disease activity. As HCQ treatment was shown to reduce the blood IFN-I signature in patients and inhibit pDC response (19, 41), this led to the hypothesis that the effect of HCQ on pDC response may be a relevant mechanism of action of the drug. The objectives of our study was to investigate the effect of HCQ therapy on pDC response to multiple TLR agonists and whether 24F4A, an anti-BDCA2 antibody (BIIB059) could add any additional benefit in modulating this response.

In this study, both *in vitro* and *in vivo* treatment with HCQ led to a reduction in IFNα production after TLR9 stimulation and did not consistently impact IFNα production from TRL7/8 agonist (R848) or TLR7 agonist (ssRNA)- stimulated pDCs. This was similar to a previous report in which pDCs from HCQ-treated patients showed a reduction of TLR9-induced IFNα response, while TLR7-induced IFNα response using imiquimod was not significantly affected (19, 20). TLR9 and TLR7 are both thought to interact with their natural ligand in the endosomes. TLR9 maturation is mediated by the cleavage of its ectodomain by cathepsin proteases and asparagine endopeptidase, which depends on the acidification of the endosome for activity (42, 43). In contrast, TLR7 maturation is mediated by furin-like protein convertases, which are active at neutral pH (44). Antimalarials such as HCQ are thought to mediate their inhibitory effect on TLR activation through increasing the endosomal pH, although some data suggest that clinically relevant concentrations may not lead to deacidification of the endosomes (18, 21, 45). The dependence of TLR9 maturation on an acidic endosomal pH may explain the more potent suppression of HCQ on TLR9 activation vs. TLR7. Another reported mechanism for HCQ inhibition on TLR activation is through binding to nucleic acids and masking their ability to interact with TLRs (45). To further explore this mechanism of action we used TLR7 agonist, ssRNA, in addition to an imidazoquinoline, R848. Note that R848 can also act as a TLR8 agonist; TLR8 induces IFN-I, but less robustly, in human pDCs (28, 46). In this study, HCQ did not consistently reduce IFNα after a nucleic acid- mediated TLR7 stimulation, confirming that HCQ is less potent at inhibiting TLR7-mediated IFNα production by pDCs as compared to TLR9. Under all TLR conditions tested, additional treatment with 24F4A was able to further reduce the residual IFNα response regardless of HCQ concentration. The cell number constraint from the PBMC collected clinically precluded a thorough dose response of 24F4A. The concentration of 24F4A (10 μg/mL) was considered to be clinically relevant as it was described to achieve complete BDCA2 internalization on human pDCs *in vitro* (23). Additionally, blood exposure of 10 μg/mL of 24F4 A also led to complete internalization of BDCA2 on circulating pDCs in treated healthy volunteers and SLE patients with CLE (33).

While both HCQ and 24F4 treatment lead inhibition of TLR-induced IFN-I from pDCs, the mechanism of action of the two drugs is likely distinct. HCQ has been speculated to inhibit TLR7,8,9 activation either by affecting the endosome pH or by inhibiting the nucleic acid binding to the TLRs (discussed above). In contrast, BDCA2 crosslinking on pDCs is thought to elicit a BCR-like signaling cascade that leads to the inhibition of IFNα production (47–50). Additionally, BDCA2 crosslinking was shown to reduce IRF7 nuclear translocation, which is a key mechanism of TLR-mediated transcription of IFNα (47). Our results showed that 24F4A leads to additional inhibitory effects on pDC responses when added to HCQ treatment, supporting the hypothesis that HCQ and 24F4A likely act through non-redundant mechanisms.

In addition to producing abundant amounts of IFN-I in response to TLR stimulation, pDCs are able to produce other proinflammatory mediators. Both HCQ and anti-BDCA2 treatment have been shown to inhibit TLR-induced proinflammatory cytokines from pDCs (e.g., TNFα and IL-6) (19, 49, 51). To explore the effect of HCQ and 24F4 on IL-6 and TNF-α, PBMC from healthy human donors were stimulated with various TLR ligands in the presence of 24F4 and HCQ alone or in combination and flow cytometry was used to determine the percentage TNFα and IL-6 positive pDCs (Supplementary Figure 5A). In general, the combination of 24F4A and HCQ reduced the percentage of pDCs positive for TNFα or IL-6 more profoundly than each alone after CpG-A and ssRNA stimulation (*p* < 0.05 for TNFα and trends for IL-6 with *p* ≤ 0.09). No significant effect was observed after R848 stimulation (Supplementary Figure 5B). Importantly, we confirmed that pDCs are the major producers of IFNα (Supplementary Figure 2) whereas other cells types can contribute to TNFα and IL-6 production in response to TLR stimulation (Supplementary Figure 5C). Based on these data, the relative contribution of pDC-derived inflammatory mediators in disease conditions, such as SLE, remains to be determined. It is possible that pDCs become an appreciable source of cytokines and chemokines in SLE target organs such as the skin, where they have been shown to accumulate (13, 14).

Although HCQ therapy is widely prescribed, its clinical use has been impeded by two factors: adverse events and low compliance of patients to treatment. The most common side effects of HCQ are mild gastrointestinal effects, skin rashes, and skin hyperpigmentation. These adverse events are believed to affect patient's compliance to treatment (52, 53). More severe but less common side effects include retinopathy, cardiomyopathy, and myopathy (53). Recent studies suggest that retinal toxicity occurs with overall prevalence of 7.5%, with frequency increasing with higher daily dose, duration of use, or compromised kidney function (54, 55). Our *in vitro* data showed that 24F4A treatment alone is able to inhibit pDC IFNα responses to TLR agonists and that the combination of 24F4A and HCQ led to a greater functional inhibition of pDCs than HCQ alone especially after ssRNA and R848 stimulation. 24F4A may be promising as an alternative treatment based on its robust inhibition of pDC responses or as an add on therapy. BIIB059 is currently being investigated in a phase II cinical trial in patients with SLE and/or CLE (NCT02847598). In one part of the study, to be enrolled, patients must have active CLE despite an adequate trial of conventional therapies which include antimalarial agents. Hence, additional information will become available on the effects of BIIB059 in patients who are taking antimalarial agents, patients who have failed to respond to antimalarial or have discontinued antimalarial agents due to side effects or poor tolerability. In the future, it might be interesting to determine if BIIB059 has HCQ-sparing potential.

In addition, blood HCQ levels in patients are often reported to be in the sub-therapeutic range (typically <500 ng/ml). Approximately 10–13% patients appear to be non-compliant with very low to undetectable blood HCQ levels and 44–77% patients show sub-therapeutic range of blood HCQ (30–32). Findings from our study were similar with 10% of patients with HCQ prescribed having undetectable or <20 ng/ml blood HCQ and 63% patients with sub-therapeutic blood HCQ levels (7 patients of the study were HCQ-naïve at the baseline visit). In this context, less frequent subcutaneous administration of a pDC specific therapy could be a unique opportunity to effectively and sustainably affect pDC responses in CLE patients. BIIB059 is currently being studied in phase II trial in CLE and SLE patients via subcutaneous monthly administration with an additional dose at week 2 (NCT02847598).

Among the antimalarial molecules, quinacrine has also been shown to provide clinical benefit in CLE in combination with HCQ with a greater effect compared to monotherapies or for the treatment of patients refractory to HCQ therapy only (56). The underlying mechanisms of this additional effect of quinacrine on HCQ therapy in CLE have been poorly studied, although it was recently reported that quinacrine, but not HCQ, may affect TNFα release from PBMC of DM and CLE patients (20). Although our study was not designed to address this question, 24F4A seemed to show an additional effect in inhibiting pDC IFN-I response in the presence of both HCQ and quinacrine. Further studies will be needed to better understand how quinacrine treatment provides an additional benefit to HCQ therapy and to confirm the additional effect of 24F4A on pDC response from patients receiving combined HCQ and quinacrine therapies.

Altogether, our study highlights that 24F4A (BIIB059), a specific pDC-targeting drug, potently inhibits pDC responses in CLE patients independent of concomitant HCQ therapy. Since the inhibition of pDCs in CLE may be a critical mechanism for providing clinical benefit, our results suggest that 24F4A could be a unique approach to specifically and robustly target pDCs in naïve CLE patients or patients on antimalarial background therapies.

## Ethics Statement

We confirm that any aspect of the work covered in this manuscript that has involved human patients has been conducted with the ethical approval of all relevant bodies. The study was performed according the Declaration of Helsinki, and the study protocol was approved by the Institutional Review Board at the University of Pennsylvania (IRB #821191). The authors have received consent forms from any participants in the study and have these forms available in case they are requested by the editor.

## Author Contributions

AG, AP, NF, VW, and DR designed the studies. VW overviewed patient enrollment. AG, AP, C-AM, RD, WW, and DD performed the experiments and/or analyzed results. AG, AP, VW, and DR wrote the main manuscript text. All the authors reviewed the manuscript.

### Conflict of Interest Statement

AG, AP, C-AM, RD, WW, DD, NF, and DR are employees Biogen Inc MA and shareholders. VW consults and has research grants from Biogen Inc MA.

## Supplementary Material

Supplementary Figure 1 Effect of HCQ alone or in combination with 24F4 on IFNα release from human whole blood after CpG-A, R848 or ssRNA stimulation. **(A)** Whole blood from human healthy patients was stimulated or not with CpG-A (10 μM with *n* = 7 donors), R848 (1 μM with *n* = 6 donors) or ssRNA (4 μg/ml with *n* = 4 donors) complexed with pARG in presence HCQ with or without 24F4 or in presence of isotype mAb control (10 μg/ml). Secreted IFNα was measured 18h after stimulation in the serum using ELISA and was plotted as percentage of secreted IFNα from TLR-stimulated with no drug condition for each donor (% of control). **(B)** Whole blood from human healthy patients was stimulated or not ssRNA (4 μg/ml) complexed with pARG in the presence of HCQ. Secreted IFNα was measured 18h after stimulation in the serum using ELISA. Panel shows mean values of IFNα from three different donors.

Supplementary Figure 2 Production of IFNα by pDCs can be detected in PBMC by flow cytometry and correlates with secreted IFNα levels measured by ELISA. **(A)** Representative dot plots from flow cytometry analyses of IFNα-producing pDCs from healthy donors PBMC using either BDCA2 (top panel) or BDCA4 and CD123 (bottom) as pDC-specific cell markers. Percents from red box represent the percent of IFNα-positive pDCs first gating on BDCA2+ cells. **(B)** Percentage of IFNα-producing pDCs detected in PBMC obtained from healthy donors upon different doses of CpG-A, R848 or ssRNA stimulation (*n* = 4 healthy donors). **(C)** Association between the percentage of IFNα-producing pDCs measured by flow cytometry and the levels of secreted IFNα measured by ELISA from the same PBMC sample stimulated with CpG-A at same doses as in **(B)** (data from 3 healthy donors, data points are color-coded based on the donor). Statistical association was assessed using Pearson's correlation.

Supplementary Figure 3 Treatment with 24F4A, but not with isotype control mAb, inhibit pDC IFNα response to CpG-A, R848, and ssRNA stimulation. Representative dot plots of IFNα+ cells within a BDCA4+ and CD123+ gate from PBMC obtained from healthy donors (*n* = 2) after CpG-A (10 μM), R848 (1 μM) or ssRNA (4 μg/ml) or without pre-treatment with 24F4A or isotype control mAb (10 μg/ml for 30 min).

Supplementary Figure 4 24F4A further reduces pDC IFNα production after CpG-A, R848 or ssRNA stimulations of PBMC isolated from CLE patients under HCQ and Quinacrine treatment. Effect of 24F4A on the percentage of IFNα-producing pDCs induced by CpG-A, R848 and ssRNA stimulations and detected by flow cytometry in PBMC from CLE patients without detectable blood HCQ, with detectable low or high HCQ level and with or without concomitant quinacrine therapy with at least 5 donors with detectable blood HCQ without quinacrine concomitant treatment and at least 8 donors with detectable blood HCQ with quinacrine concomitant treatment. Statistical significance was assessed with a two-tailed paired Student's *t*-test using log2-transformed values (^*^*p* < 0.05, ^**^*p* < 0.01).

Supplementary Figure 5 24F4A reduces pDC TNFα and IL-6 production after CpG-A and ssRNA, but not R848, stimulations of PBMC isolated from healthy donors similarly as or better than HCQ treatment. Whole blood samples from healthy donors were treated with HCQ (1000 ng/ml) or not for 1 h prior to PBMC isolation and then stimulated with CpG-A (10 μM), R848 (1 μM) or ssRNA (4 μg/ml) for 6h. PDCs were identified as BDCA4+ and CD123+ positive. **(A)** Representative flow cytometry plot for TNFα and IL-6 intracellular stainings in pDCs. **(B)** Percentages of TNFα or IL-6 -producing pDCs induced by CpG-A, R848 and ssRNA stimulations after pre-treatment HCQ or mAbs (*n* = 6 healthy donors). Statistical significance was assessed with a two-tailed paired Student's *t*-test (ns, non-significant *p* ≥ 0.05, ^*^*p* < 0.05, ^**^*p* < 0.01, ^****^*p* < 0.0001). **(C)** Representative flow cytometry dot plots for TNFα and IL-6 intracellular stainings of PBMC excluding the pDCs (from *n* = 6 healthy donors).

Supplementary Table 1 Effect of 24F4A on pDC IFNs production after CpG-A or R848 stimulations of PBMC isolated from CLE patients regardless blood HCQ levels.
