# Supplementary figures and images for: Effect of in vivo Hydroxychloroquine and ex vivo Anti-BDCA2 mAb Treatment on pDC IFNα Production From Patients Affected With Cutaneous Lupus Erythematosus
Source: Front Immunol. 2019 Feb 21;10:275. doi: 10.3389/fimmu.2019.00275 (PMC6394354; doi:10.3389/fimmu.2019.00275)

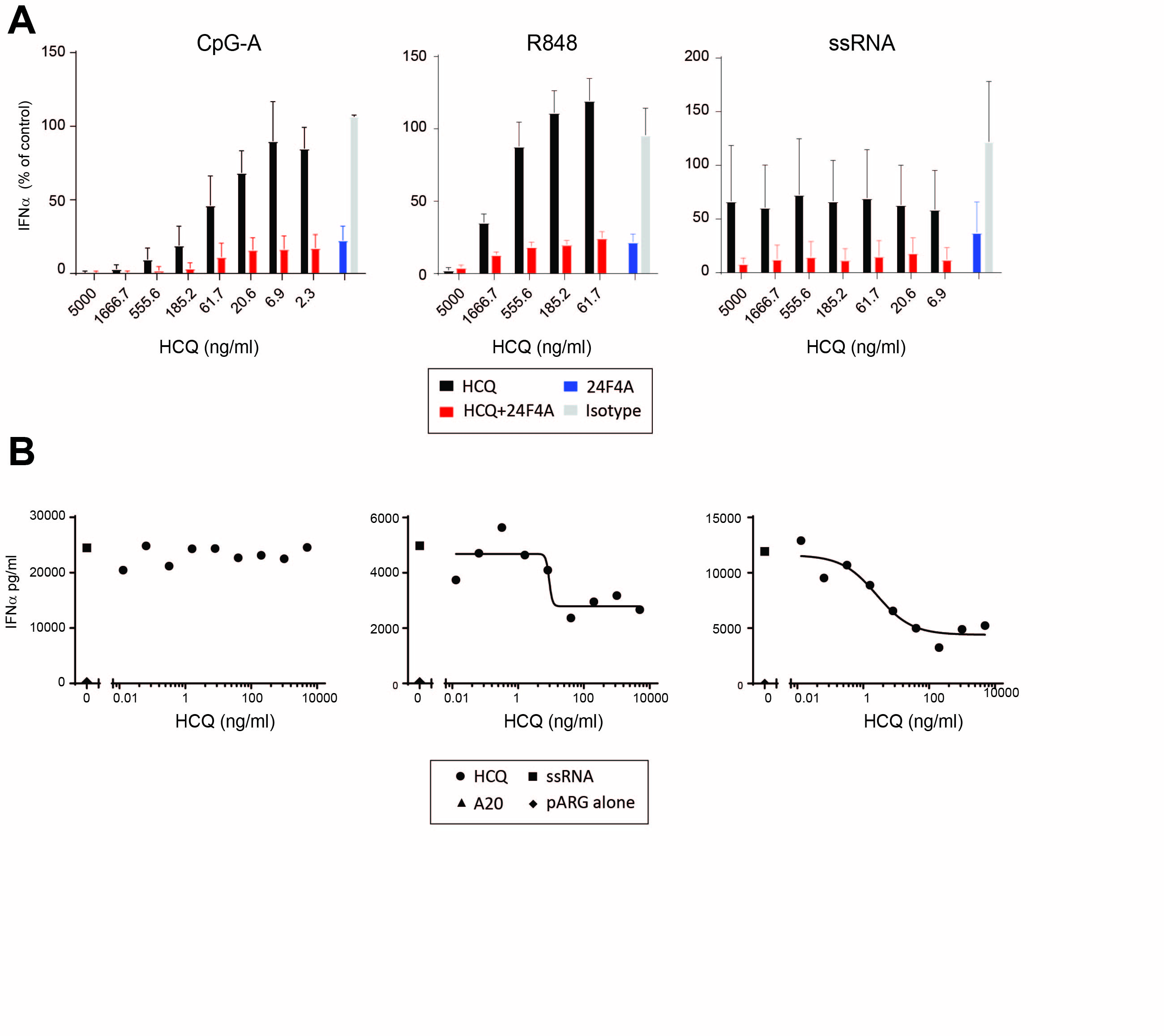

Supplement: Supplementary Figure 1 — Effect of HCQ alone or in combination with 24F4 on IFNα release from human whole blood after CpG-A, R848 or ssRNA stimulation. (A) Whole blood from human healthy patients was stimulated or not with CpG-A (10 μM with n = 7 donors), R848 (1 μM with n = 6 donors) or ssRNA (4 μg/ml with n = 4 donors) complexed with pARG in presence HCQ with or without 24F4 or in presence of isotype mAb control (10 μg/ml). Secreted IFNα was measured 18h after stimulation in the serum using ELISA and was plotted as percentage of secreted IFNα from TLR-stimulated with no drug condition for each donor (% of control). (B) Whole blood from human healthy patients was stimulated or not ssRNA (4 μg/ml) complexed with pARG in the presence of HCQ. Secreted IFNα was measured 18h after stimulation in the serum using ELISA. Panel shows mean values of IFNα from three different donors. [file Image_1.TIF]

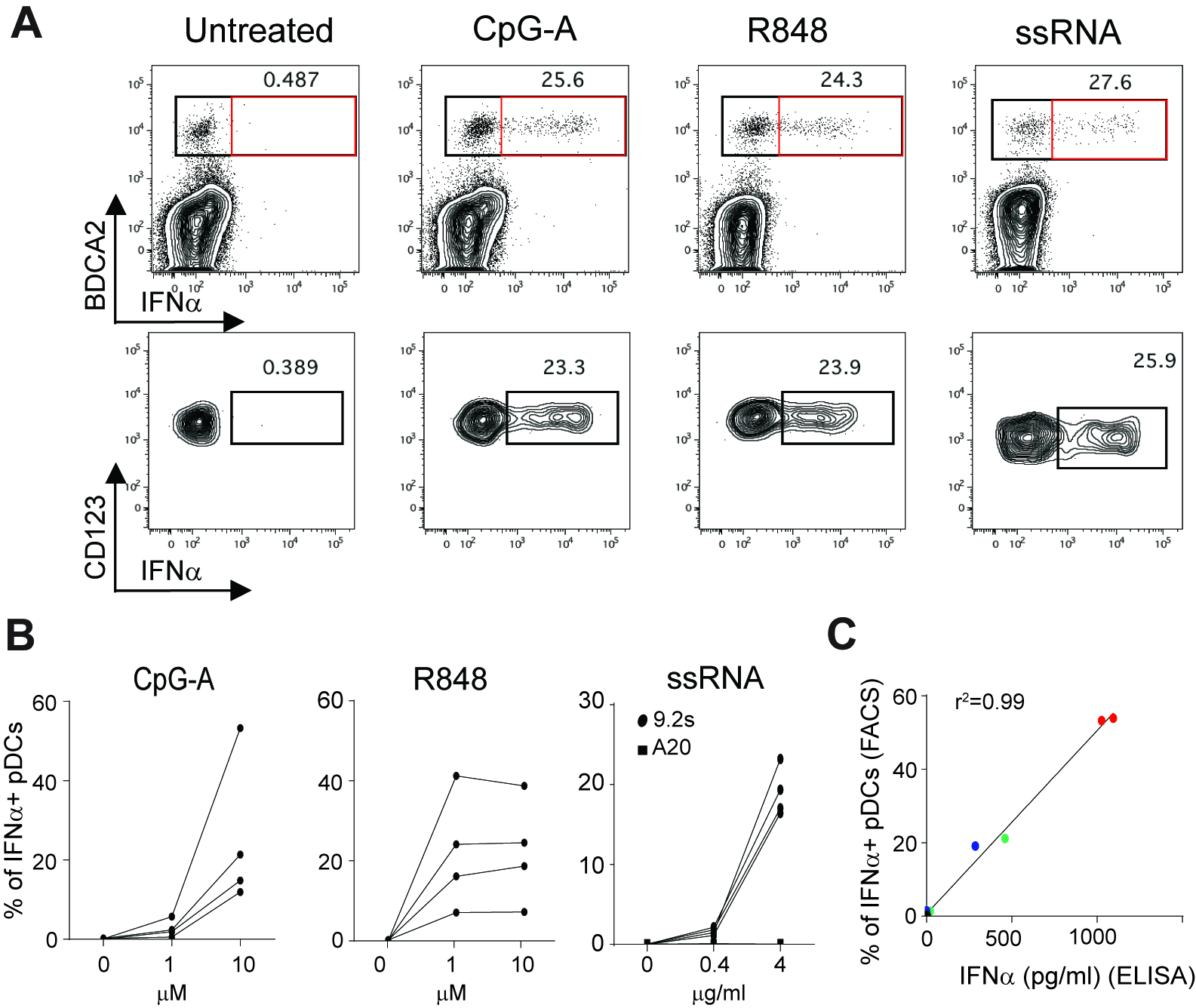

Supplement: Supplementary Figure 2 — Production of IFNα by pDCs can be detected in PBMC by flow cytometry and correlates with secreted IFNα levels measured by ELISA. (A) Representative dot plots from flow cytometry analyses of IFNα-producing pDCs from healthy donors PBMC using either BDCA2 (top panel) or BDCA4 and CD123 (bottom) as pDC-specific cell markers. Percents from red box represent the percent of IFNα-positive pDCs first gating on BDCA2+ cells. (B) Percentage of IFNα-producing pDCs detected in PBMC obtained from healthy donors upon different doses of CpG-A, R848 or ssRNA stimulation (n = 4 healthy donors). (C) Association between the percentage of IFNα-producing pDCs measured by flow cytometry and the levels of secreted IFNα measured by ELISA from the same PBMC sample stimulated with CpG-A at same doses as in (B) (data from 3 healthy donors, data points are color-coded based on the donor). Statistical association was assessed using Pearson's correlation. [file Image_2.TIF]

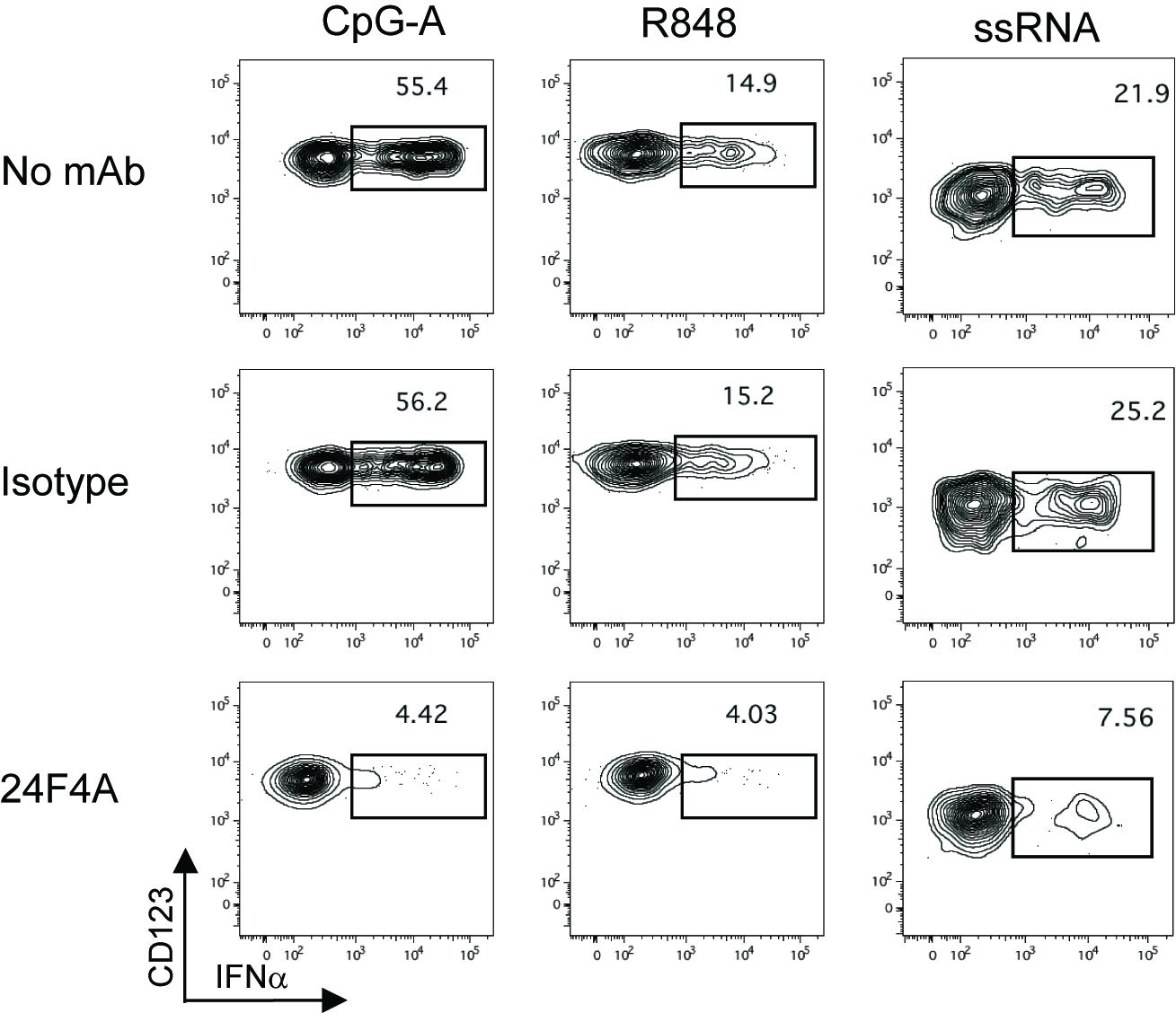

Supplement: Supplementary Figure 3 — Treatment with 24F4A, but not with isotype control mAb, inhibit pDC IFNα response to CpG-A, R848, and ssRNA stimulation. Representative dot plots of IFNα+ cells within a BDCA4+ and CD123+ gate from PBMC obtained from healthy donors (n = 2) after CpG-A (10 μM), R848 (1 μM) or ssRNA (4 μg/ml) or without pre-treatment with 24F4A or isotype control mAb (10 μg/ml for 30 min). [file Image_3.TIF]

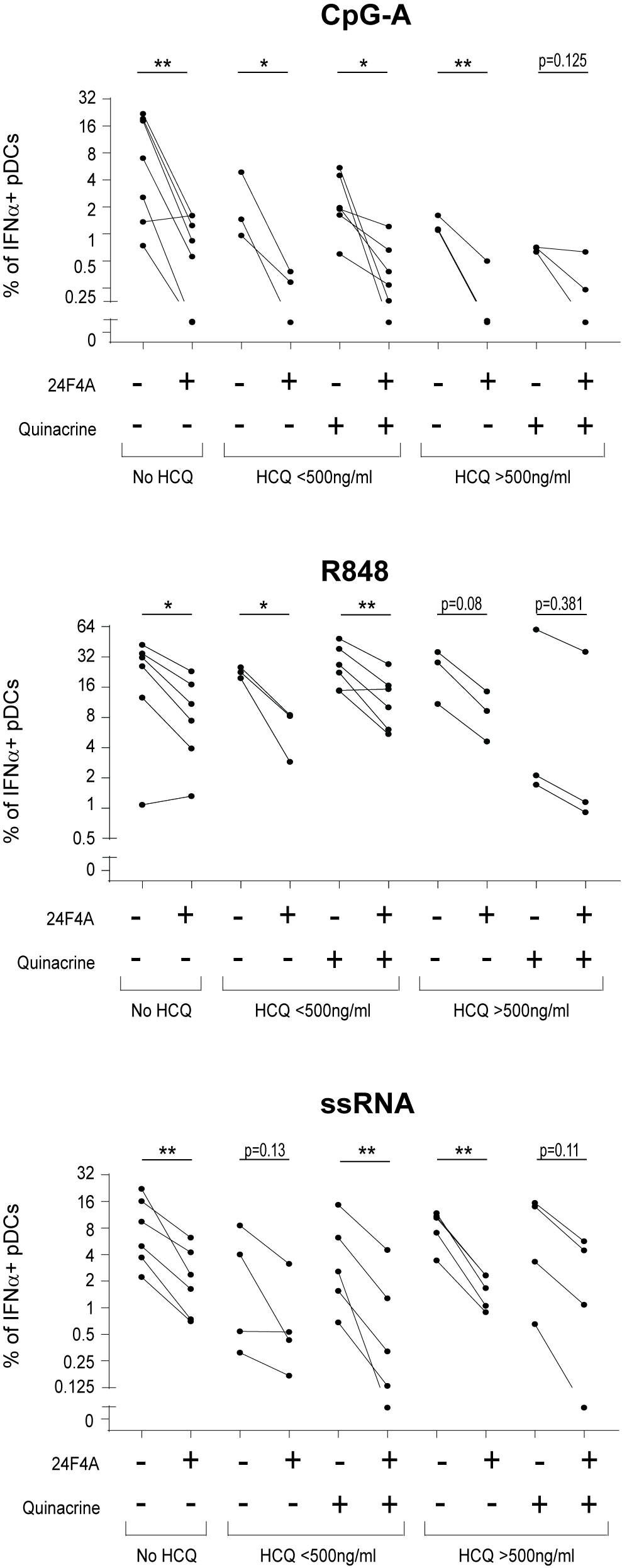

Supplement: Supplementary Figure 4 — 24F4A further reduces pDC IFNα production after CpG-A, R848 or ssRNA stimulations of PBMC isolated from CLE patients under HCQ and Quinacrine treatment. Effect of 24F4A on the percentage of IFNα-producing pDCs induced by CpG-A, R848 and ssRNA stimulations and detected by flow cytometry in PBMC from CLE patients without detectable blood HCQ, with detectable low or high HCQ level and with or without concomitant quinacrine therapy with at least 5 donors with detectable blood HCQ without quinacrine concomitant treatment and at least 8 donors with detectable blood HCQ with quinacrine concomitant treatment. Statistical significance was assessed with a two-tailed paired Student's t-test using log2-transformed values (*p < 0.05, **p < 0.01). [file Image_4.TIF]

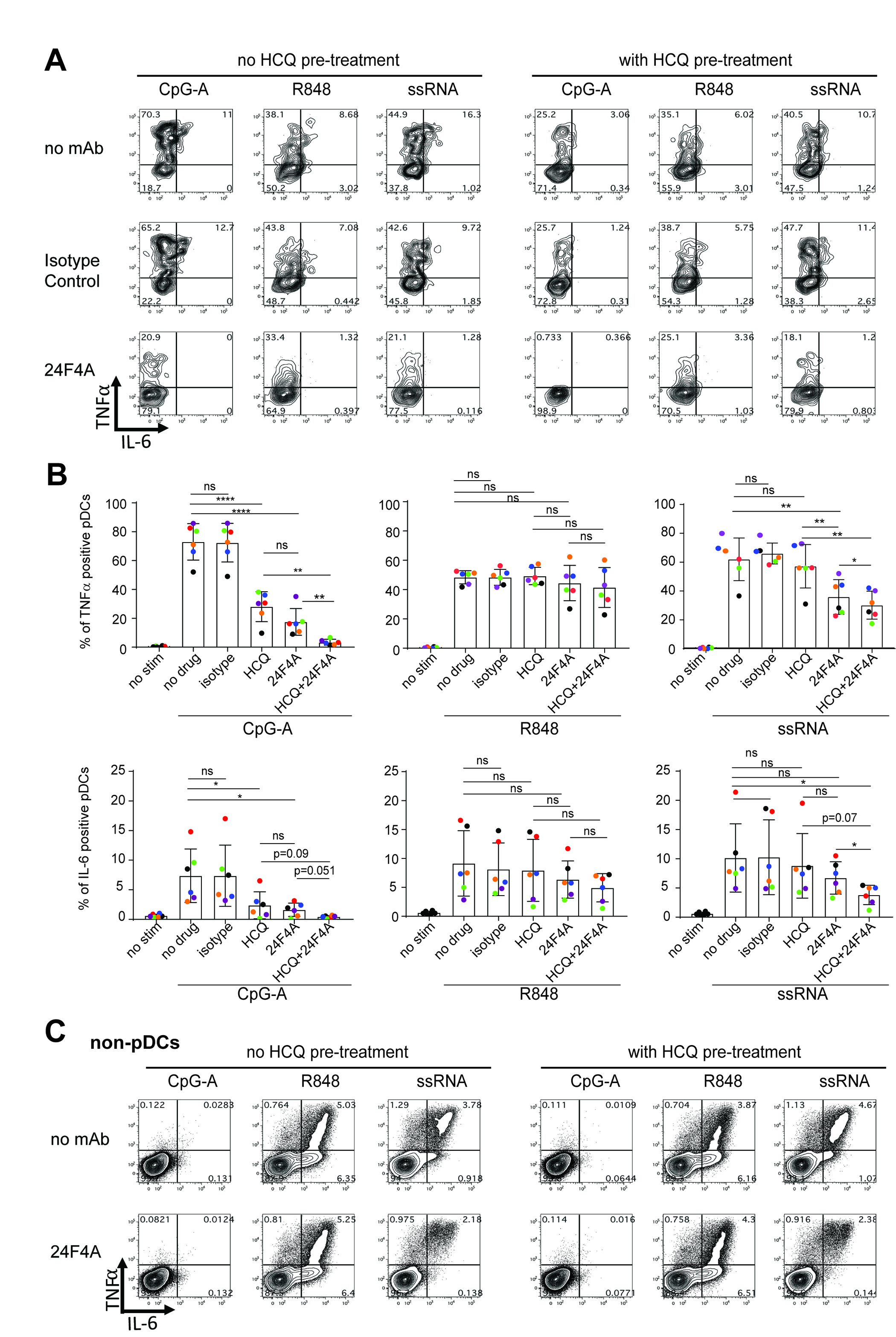

Supplement: Supplementary Figure 5 — 24F4A reduces pDC TNFα and IL-6 production after CpG-A and ssRNA, but not R848, stimulations of PBMC isolated from healthy donors similarly as or better than HCQ treatment. Whole blood samples from healthy donors were treated with HCQ (1000 ng/ml) or not for 1 h prior to PBMC isolation and then stimulated with CpG-A (10 μM), R848 (1 μM) or ssRNA (4 μg/ml) for 6h. PDCs were identified as BDCA4+ and CD123+ positive. (A) Representative flow cytometry plot for TNFα and IL-6 intracellular stainings in pDCs. (B) Percentages of TNFα or IL-6 -producing pDCs induced by CpG-A, R848 and ssRNA stimulations after pre-treatment HCQ or mAbs (n = 6 healthy donors). Statistical significance was assessed with a two-tailed paired Student's t-test (ns, non-significant p ≥ 0.05, *p < 0.05, **p < 0.01, ****p < 0.0001). (C) Representative flow cytometry dot plots for TNFα and IL-6 intracellular stainings of PBMC excluding the pDCs (from n = 6 healthy donors). [file Image_5.TIF]
